# PhenoRank: reducing study bias in gene prioritization through simulation

**DOI:** 10.1093/bioinformatics/bty028

**Published:** 2018-01-17

**Authors:** Alex J Cornish, Alessia David, Michael J E Sternberg

**Affiliations:** Department of Life Sciences, Center of Bioinformatics and Systems Biology, Imperial College London, London, UK

## Abstract

**Motivation:**

Genome-wide association studies have identified thousands of loci associated with human disease, but identifying the causal genes at these loci is often difficult. Several methods prioritize genes most likely to be disease causing through the integration of biological data, including protein–protein interaction and phenotypic data. Data availability is not the same for all genes however, potentially influencing the performance of these methods.

**Results:**

We demonstrate that whilst disease genes tend to be associated with greater numbers of data, this may be at least partially a result of them being better studied. With this observation we develop PhenoRank, which prioritizes disease genes whilst avoiding being biased towards genes with more available data. Bias is avoided by comparing gene scores generated for the query disease against gene scores generated using simulated sets of phenotype terms, which ensures that differences in data availability do not affect the ranking of genes. We demonstrate that whilst existing prioritization methods are biased by data availability, PhenoRank is not similarly biased. Avoiding this bias allows PhenoRank to effectively prioritize genes with fewer available data and improves its overall performance. PhenoRank outperforms three available prioritization methods in cross-validation (PhenoRank area under receiver operating characteristic curve [AUC]=0.89, DADA AUC = 0.87, EXOMISER AUC = 0.71, PRINCE AUC = 0.83, *P *< 2.2 × 10^−16^).

**Availability and implementation:**

PhenoRank is freely available for download at https://github.com/alexjcornish/PhenoRank.

**Supplementary information:**

Supplementary data are available at *Bioinformatics* online.

## 1 Introduction

Genome-wide association studies (GWAS) have identified thousands of genomic variants associated with a range of human traits, including susceptibilities to many diseases. Disease-associated variants are themselves rarely causal and instead ‘tag’ regions of the genome containing variants in linkage disequilibrium, any one of which may be the causal variant. These causal variants may be located in the coding region of a gene, or in a regulatory region and disrupt the expression of a gene through *cis* or *trans*-acting regulatory mechanisms (Jäger *et al.*, 2015), making the identification of causal genes often difficult. This has led to the development of methods that integrate biological data to prioritize likely causal genes (Erten *et al.*, 2011; Köhler *et al.*, 2008; Smedley *et al.*, 2015; Vanunu *et al.*, 2010; Yates *et al.*, 2014).

Network-based methods have been demonstrated to be effective at prioritizing disease-causing genes (Cowen *et al.*, 2017; Erten *et al.*, 2011; Köhler *et al.*, 2008; Smedley *et al.*, 2015; Vanunu *et al.*, 2010; Yates *et al.*, 2014). These approaches often score genes more highly if they, or their protein products, interact with genes known to be associated with the query disease, or genes associated with diseases that are phenotypically similar to the query disease (Erten *et al.*, 2011; Smedley *et al.*, 2015; Vanunu *et al.*, 2010). Yates *et al.* found that disease proteins tend to occupy more central positions in PPI networks than non-disease proteins (Yates and Sternberg, 2013), whilst Das *et al.* found this to be true, but only for PPI networks generated through literature-curation (Das and Yu, 2012). Some network-based methods therefore score genes and gene variants whose protein products are more central in PPI networks higher than those that are less central (Fu *et al.*, 2014; Yates *et al.*, 2014). The centrality measures used by these methods are correlated with the number of interactions a protein is involved in (Valente *et al.*, 2008). It has been suggested however that some proteins may be involved in more interactions in literature-curated PPI networks as a result of them being better studied (Das and Yu, 2012; Gillis and Pavlidis, 2012). If this is true, then network-based methods that score genes central in a network more highly may be less effective at prioritizing genes that are less well studied, as these genes may be more peripheral in a network, as a result of them having fewer available data.

Databases such as ClinVar (Landrum *et al.*, 2016), OMIM (Amberger *et al.*, 2015) and UniProtKB (The UniProt Consortium, 2014) collate data on the relationships between genetic variation and human disease. Databases that associate genetic variation with phenotypic abnormalities have also been established for model organisms (Bult *et al.*, 2016) and used to study human disease (Chen *et al.*, 2012; Smedley *et al.*, 2015). It has been demonstrated that disease genes can be prioritized by identifying genes implicated in phenotypically similar diseases (Smedley *et al.*, 2015; Vanunu *et al.*, 2010). For example, novel causal genes for prostate cancer may be inferred by identifying genes implicated in other cancers (Vanunu *et al.*, 2010). Similarly, candidate disease genes in humans can be prioritized by identifying orthologous mouse genes whose mutation causes similar phenotypes in mice (Chen *et al.*, 2012; Smedley *et al.*, 2015).

Multiple approaches have been proposed to quantify the phenotypic similarity of human diseases. Phenotype ontologies, such as the Human Phenotype Ontology (HPO) (Köhler *et al.*, 2014) and the Mammalian Phenotype Ontology (MP) (Smith *et al.*, 2005), provide standardized and structured vocabularies of observed phenotypic abnormalities. Multiple phenotype ontology terms can be mapped to a human disease to describe the phenotypic features of the disease. These features can include abnormalities associated with the disease (for example ‘Abnormality of the outer ear’), its mode of inheritance (for example ‘Autosomal dominant inheritance’) and clinical features (for example ‘Childhood onset’). The structured nature of ontologies allows the similarity of terms to be quantified. For example, the HPO terms ‘IgM deficiency’ and ‘IgE deficiency’ are both subclasses of ‘Decreased antibody level in blood’ and may therefore be considered similar. The HPO terms ‘IgM deficiency’ and ‘Dementia’ are less well connected in the ontology and may therefore be considered less similar. Semantic similarity methods such as simGIC measure the similarity of sets of terms in ontologies (Pesquita *et al.*, 2008) and can therefore quantify the similarity of sets of phenotype terms annotating human diseases and mouse mutants, thereby providing a measure of their phenotypic similarity. It has been demonstrated however that semantic similarity methods can be biased by data availability. For example, the simGIC method tends to identify larger sets of terms, sets of terms that are more similar in size, and sets of terms from deeper ontology levels, as being more similar (Kulmanov and Hoehndorf, 2017). Gene prioritization methods that use semantic similarity to quantify phenotypic similarity may therefore be biased by the numbers of phenotype terms annotating human diseases and model organism mutants, which may reflect how well studied these entities are.

In this study, we demonstrate that whilst disease genes are involved in greater numbers of PPIs than non-disease genes in some PPI databases, this may be at least partly a result of them being better studied. Scoring genes with more available data more highly may reduce the ability of a method to prioritize less-well-studied genes, for which fewer data are likely to be available. We therefore develop PhenoRank, which uses PPI and phenotype data from multiple species to prioritize disease genes, whilst avoiding being biased by the number of data associated with each gene. Bias is avoided by comparing gene scores generated for the query disease against gene scores generated using simulated sets of phenotype terms. Using this simulation-based approach ensures PhenoRank is not biased towards genes with more available data and improves its performance.

## 2 Materials and methods

### 2.1 PPI data

PPI data were downloaded from four databases: BioGRID (version 3.4.131) (Chatr-Aryamontri *et al.*, 2015), HI-II-14 (on 27 November, 2015) (Rolland *et al.*, 2014), HPRD (on 30 March, 2015) (Keshava Prasad *et al.*, 2009) and IntAct (on 4 January, 2016) (Orchard *et al.*, 2014). Only direct interactions, associations and physical associations were obtained from BioGRID and IntAct. Duplicate interactions, looping interactions and interactions that did not occur between two *H.sapiens* proteins were excluded. Some PPI resources do not record interactions between different protein isoforms and we therefore considered all interactions at the gene level. Combined data from the four resources, containing 210 914 unique interactions spanning 16 184 genes, were used in PhenoRank (Supplementary Table S1).

### 2.2 Human disease variant data

Data downloaded from ClinVar (on 22 October, 2016), OMIM (on 1 November, 2016) and UniProtKB (on 22 October, 2016) were used to define the disease–gene associations used in PhenoRank. ClinVar variants not marked as pathogenic or likely pathogenic, or whose review status was less than two stars were excluded. Non-disease variants from UniProtKB were excluded. Disease–gene associations from OMIM were not considered if the molecular basis of the disease is unknown. Diseases reported using vocabularies other than OMIM were mapped to OMIM terms using the cross-referencing provided by the Disease Ontology (DO) (Kibbe *et al.*, 2015). Using these data, we define a disease as being associated with a gene if a gene variant is reported as being disease causing. The combined dataset contains 5685 unique associations between 4729 diseases and 3713 genes (Supplementary Table S2).

### 2.3 Mouse phenotype data

Genotypes and phenotype term annotations for 24 834 mouse mutants and human-mouse gene orthology data were downloaded from the Mouse Genomics Database (MGD, on 13 October, 2016). Human orthologs of the mutated gene in 21 143 mouse mutants were identified using the orthology data.

### 2.4 Annotating diseases with phenotype terms

Mappings between disease terms (from OMIM) and phenotype terms (from HPO and MP) from HPO (Köhler *et al.*, 2014) and Hoehndorf *et al.* (2015) are used in PhenoRank to measure the phenotypic similarity of the query disease and diseases in OMIM. Hoehndorf *et al.* mapped phenotype terms (from HPO and MP) to disease terms (from DO) through automated text mining. Hoehndorf *et al.* determined that the 21 phenotype terms most strongly associated with each disease were most informative when quantifying phenotypic similarity and we therefore include these phenotype term mappings in PhenoRank. The cross-referencing provided by the DO was used to transfer the mapped phenotype terms to the corresponding OMIM diseases. The combined dataset contains 128 695 unique mappings between 7042 OMIM diseases and 8313 unique HPO and MP phenotype terms (Supplementary Table S3).

### 2.5 Measuring phenotypic similarity

PhenoRank uses the simGIC similarity measure to compute the phenotypic similarity of human diseases and mouse mutants (Supplementary Fig. S1). Let *W_i_* and *W_j_* be two sets of phenotype ontology terms. In our case, these phenotype terms are terms from the HPO or MP that either annotate a disease or describe abnormalities observed in a mouse mutant (Supplementary Fig. S2). Uberpheno, a cross-species ontology generated by integrating multiple phenotype ontologies, including the HPO and MP, is used to compare sets of terms (Köhler *et al.*, 2013). The true path rule states that association with an ontology term implies association with all ancestors of the term (Pesquita *et al.*, 2008), and we therefore add the ancestors of each term in *W_i_*and *W_j_* to the respective set using Uberpheno. Each term in Uberpheno is weighted by its information content (IC), defined as the negative logarithm of the probability that a given disease or mouse mutant is annotated with the term. Using simGIC, the similarity *S* of *W_i_* and *W_j_* is the Jaccard similarity coefficient weighted by the IC of each term:

$$S\left(W_{i},W_{j}\right)=\frac{\sum_{x\in W_{i}\cap W_{j}}IC(x)}{\sum_{y\in W_{i}\cup W_{j}}IC(y)}$$

### 2.6 Disease gene prioritization using PhenoRank

**Fig. 1.**
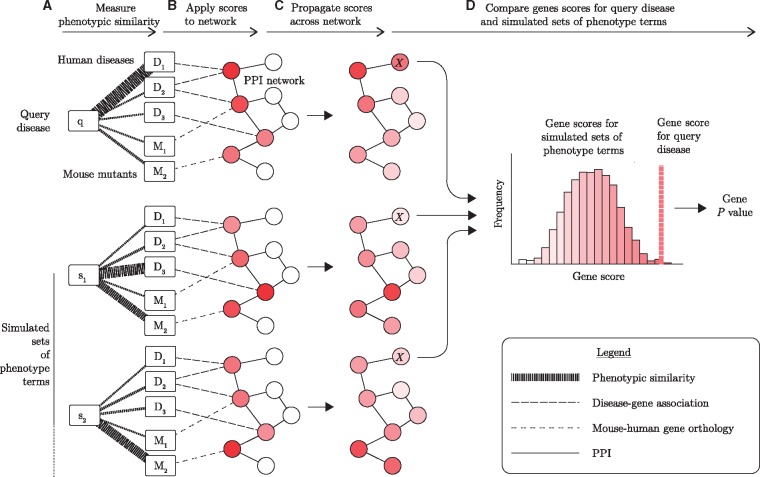
PhenoRank overview. (**A**) Phenotypic similarity of the query disease (q) to each disease in OMIM (D) and each mouse mutant in MGD (M) is quantified. Thicker lines represent stronger phenotypic similarities. (**B**) Phenotypic similarity scores are applied to genes in a PPI network, using known disease–gene associations and mouse-human gene orthology data. Darker nodes represent genes with greater relevance to the disease of interest. (**C**) Phenotypic relevance scores are propagated across a PPI network, so that genes that interact with many high scoring genes are also scored highly. (**D**) Gene scores generated for the query disease are compared against gene scores generated using simulated sets of phenotype terms (S). By comparing the score the gene receives for the query disease against the distribution of scores the gene receives for the simulated sets of phenotype terms, a *P* value for each gene is generated. In this illustrative example, the computation of a *P* value for the gene marked X is shown. While only two simulated sets of disease phenotypes are shown, PhenoRank is run with 1000 simulated sets by default

Let *D* be the diseases represented in ClinVar, OMIM and UniProtKB, *M* be the mouse mutants reported in the MGD, *q* be the query disease so that *q*∈*D*, and *W_i_* be the set of phenotype terms mapped to phenotype data source *i*, which can be either a human disease or mouse mutant. In PhenoRank, all diseases in *D* and mouse mutants in *M* are first scored by their phenotypic similarity to query disease *q* (Fig. 1A). Phenotypic similarity is measured by comparing the ontological similarity of the set of phenotype terms mapped to *q*, to the sets of phenotype terms mapped to each disease in *D* and mouse mutant in *M*, using the simGIC method. Diseases and mouse mutants are therefore scored as being phenotypically similar to *q* if they are mapped to phenotype terms that are closely related in Uberpheno. 

These phenotypic similarity scores are used to score each gene in a PPI network*.* Let *G* = (*V*, *E*) be this network, with *V* being nodes representing genes and *E* being edges representing physical interactions between the protein products of the genes. Gene scores are computed using disease–gene associations from ClinVar, OMIM and UniProtKB, and human-mouse orthology data from the MGD (Fig. 1B). The score of gene *i* is defined as the sum of the phenotypic similarity of each associated disease and each mutant of an orthologous mouse gene, to *q*, divided by the numbers of associated diseases and mouse mutants:

$$Q_{i}=\;\frac{\sum_{j\in Y_{i}}S(W_{q},W_{j})}{\left|Y_{i}\right|}+\;\frac{\sum_{k\in Z_{i}}S(W_{q},W_{k})}{\left|Z_{i}\right|}$$

where *Y_i_*is the set of diseases associated with gene *i*, Z*_i_*is the set of mutants of mouse genes orthologous to gene *i* and *W_q_*is the set of phenotype terms annotating *q*. Genes are therefore scored highly if they are associated with a disease that is phenotypically similar to *q*, or if they are orthologous to a mouse gene whose mutation produces phenotypic abnormalities similar to *q*.

These gene scores are next propagated across *G* using the random walk with restart (RWR) method (Fig. 1C), as this approach has been shown to be effective when prioritizing disease genes and variants using network data (Köhler *et al.*, 2008; Vanunu *et al.*, 2010). Propagation of gene scores ensures that genes that interact with many genes that are phenotypically relevant to the query disease are also scored highly.

To account for the differing availability of data between genes, gene scores generated for the query disease are compared against gene scores generated using simulated sets of phenotype terms (Fig. 1D). Simulated sets of phenotype terms are generated by sampling from phenotype terms mapped to the same diseases by HPO and Hoehndorf *et al.* (Supplementary Fig. S3B), to ensure that the simulated sets of terms closely resemble the sets of phenotype terms mapped to real diseases. Sets of phenotype terms equal in size to the set of terms mapped to *q* are simulated. To simulate a set of terms of size |*W_q_*|, a single seed term is first sampled from HPO or MP. All phenotype terms in HPO and MP are then ranked by the number of times they are mapped to the same disease as the seed term, with ties ordered randomly. The seed term is itself included in this ranking. If fewer than |*W_q_*| terms are mapped to the same disease as the seed term, then a new seed term is sampled. The top |*W_q_*| ranked terms are used as the simulated set of phenotype terms, ensuring that the simulated sets contain terms that are frequently mapped to the same disease.

For each simulated set of phenotype terms, all genes are rescored using the simulated set of terms in place of the query disease, and these scores again propagated across the PPI network. When rescoring genes all data are unchanged, ensuring that the effect of data availability on each gene score is the same for the query disease and each simulated set of phenotype terms. Comparing the gene scores generated using the query disease against the gene scores generated using each simulated set of phenotype terms therefore allows differences in data availability to be negated. An empirical *P* value is computed for each gene by taking the proportion of simulated sets of phenotype terms in which the gene is scored higher than when *q* is considered. These *P* values represent the probability of observing a gene score at least as great as that observed, given that the gene is not associated with the query disease. A minimum *P* value of 1/u, where u is the number of simulated sets of phenotype terms used, is applied to ensure that no *P* values equal zero. We run PhenoRank using 1000 simulated sets of phenotype terms and use these *P* values to prioritize candidate genes. Through the application of PhenoRank to 100 randomly selected diseases, we demonstrate that PhenoRank correctly controls the type-1 error rate (Supplementary Fig. S4). PhenoRank data are available to download (https://github.com/alexjcornish/PhenoRank_Data).

### 2.7 Propagating scores across PPI networks

PhenoRank propagates gene scores across network *G* using the RWR method (Supplementary Fig. S5) (Köhler *et al.*, 2008). Let *n* be the number of vertices in *G*, *A* be the column-normalized adjacency matrix of *G* and *Q^t^* be a vector of length *n* and the distribution of scores across vertices at time *t*. *Q^0^* is the initial distribution of gene scores. The distribution of scores across time points is computed iteratively:

$$Q^{t+1}=\left(1-r\right)AQ^{t}+\;rQ^{0}$$

where *r* is the restart probability. To make these scores comparable across the query disease and the simulated sets of phenotype terms, a fixed number of iterations are completed and scores ranked before PhenoRank computes *P* values.

### 2.8 Evaluating method bias

We measured the correlation between the gene scores computed by PhenoRank and three published gene prioritization methods [DADA (Erten *et al.*, 2011), EXOMISER (Smedley *et al.*, 2015) and PRINCE (Vanunu *et al.*, 2010)] and the numbers of data associated with each gene, to determine whether the methods are biased towards genes with more available data. PhenoRank was run with and without simulated sets of phenotype terms (we refer to these method versions as PhenoRank-Simulation and PhenoRank-NoSimulation), to establish whether the use of these simulated sets of terms affects how biased PhenoRank is. EXOMISER prioritizes disease genes and variants by combining a gene-based scoring method, which uses PPI data and phenotype data from multiple species, with variant-based pathogenicity prediction. We use the gene-level scores produced by EXOMISER when evaluating performance and therefore refer to the method as EXOMISER-Walker for clarity. Gene scores were generated by applying each method to 200 diseases, randomly selected from those diseases than can be considered by all methods. We considered how five features of the data used by each method correlate with the computed gene scores:

- Network degree of each gene.
- Number of sources of phenotype data. This is defined as the number of human diseases and (if used by the method) model organism mutants associated with each gene.
- Number of annotating phenotype terms. This is defined as the median number of phenotype terms annotating the sources of phenotype data associated with each gene.
- Difference in the number of annotating phenotype terms. This is defined as the median absolute difference between the number of phenotype terms annotating the query disease, and the numbers of phenotype terms annotating each source of phenotype data associated with each gene.
- Ontology depth of annotating phenotype terms. This is defined as the median of the maximum ontology depth of the phenotype terms annotating the sources of phenotype data associated with each gene.

PhenoRank and EXOMISER-Walker both use phenotype terms annotating each source of phenotype data to measure phenotypic similarity, whilst DADA and PRINCE measure phenotypic similarity using text mining. We therefore test all five data features when considering PhenoRank and EXOMISER-Walker, but restrict our analysis to data features (i) and (ii) when considering DADA and PRINCE.

### 2.9 Evaluating method performance

The performance of each gene prioritization method was evaluated using leave-one-out cross-validation. To ensure that any observed performance differences were a result of methodology, rather than the data releases used, we ran DADA, EXOMISER-Walker and PRINCE using the same disease–gene association data used by PhenoRank. Gene-phenotype associations used by EXOMISER-Walker were also updated and the IC of each phenotype term recalculated. HPO terms mapped to OMIM diseases by the HPO were used as input when running EXOMISER-Walker. We used our own implementation of the PRINCE algorithm, which is available in the PhenoRank package.

Leave-one-out cross-validation was completed using a set of 2708 associations between diseases that can be input into all four methods, and genes that can be scored by all four methods. In each cross-validation trial, an association from this set (between disease *D_i_* and gene *g_j_*∈ *S_i_*) was masked (i.e. removed from the data used by each method). Each method was then run using disease *D_i_* as input. All other genes associated with disease *D_i_*(*g_k_*∈ *S_i_, k ≠ j*) and genes not scored by all four methods were excluded from the results. The score of gene *g_j_* relative to the scores of the other genes in the results was used to evaluate the performance of each method. This process was repeated for each of the 2708 disease–gene associations. Receiver operating characteristic (ROC) curves and the areas under these curves (AUCs) were computed using the pROC R package (Robin *et al.*, 2011).

### 2.10 Selecting method parameters

Scores propagated across a network using the RWR algorithm converge on a steady-state distribution (Cowen *et al.*, 2017). To determine the number of RWR algorithm iterations required by PhenoRank for convergence, we ran PhenoRank using 200 randomly selected OMIM terms and calculated the mean absolute differences between the gene scores computed using between 1 and 29 iterations, and the gene scores computed using 30 iterations, demonstrating that scores converge and that the mean absolute change in gene score after 20 iterations is <10^−5^ for all tested parameters (Supplementary Fig. S6A). We next conducted leave-one-out cross-validation to select an optimal value for restart probability *r*, using only the 2977 disease–gene associations reported by ClinVar, OMIM or UniProtKB that were not in the set of 2708 associations used in performance evaluation. This ensured that parameter selection and performance evaluation were independent, therefore avoiding circularity. PhenoRank performs optimally when *r *=* *0.1 (Supplementary Table S4) and we therefore ran PhenoRank using 20 iterations and *r *=* *0.1. We used the same approach to determine the number of RWR algorithm iterations required by PRINCE and select optimal values for the two PRINCE parameters (*α* and *c*). Convergence is achieved by 20 iterations (Supplementary Fig. S6B) and performance is optimal when *α * = 0.5 and *c* = −15 (Supplementary Table S4), and we therefore ran PRINCE using these values.

### 2.11 Disease classes

A disease class was identified for each OMIM disease using the ontological structure of the DO (Supplementary Table S5). Each OMIM disease was first mapped to a DO term using the cross references provided by the DO. Ancestors of these DO terms at the third level of the DO were then identified and these broader disease definitions used as disease classes. If an OMIM disease mapped to multiple third-level DO terms, then the third-level DO term mapped to the greatest number of OMIM diseases was used to classify the disease, to reduce the number of classes considered.

## 3 Results

### 3.1 Study bias in PPI databases

We analyzed the numbers of PPIs involving disease and non-disease genes to determine whether disease genes are involved in greater numbers of PPIs than non-disease genes, and whether study bias is likely to contribute to any differences. PPI data were downloaded from BioGRID, HI-II-14, HPRD and IntAct and disease–gene association data were obtained from ClinVar, OMIM and UniProtKB. If a gene is reported as being disease associated by at least one resource, then it is defined as a disease gene. Otherwise it is defined as a non-disease gene.

BioGRID, HPRD and IntAct contain PPIs curated from the literature. The proteins screened in the studies contributing to these resources depend on the aims of the studies and the generation of these data was therefore hypothesis-driven (HD). Conversely, HI-II-14 contains interactions identified in a single unbiased screen of 14 000 proteins (Rolland *et al.*, 2014) and the generation of these data was therefore hypothesis-free (HF). If disease genes are truly involved in greater numbers of PPIs than non-disease genes, we would expect disease genes to be involved in greater numbers of PPIs than non-disease genes in datasets generated using both HD and HF approaches. However, whilst disease genes are involved in greater numbers of PPIs than non-disease genes in each of the HD datasets, disease genes are involved in similar numbers of PPIs as non-disease genes in the HF dataset (Table 1).

**Table 1.** Numbers of PPIs disease and non-disease genes are involved in

Database	Disease genes		Non-disease genes		Difference
	Mean n. PPIs	Median n. PPIs	Mean n. PPIs	Median n. PPIs	
BioGRID	26.3	10.0	18.5	8.0	*P* < 2.2 × 10^−16^
HI-II-14	5.7	2.0	6.7	2.0	*P* = 0.685
HPRD	10.7	5.0	7.2	3.0	*P* < 2.2 × 10^−16^
IntAct	18.8	7.0	14.3	6.0	*P *<* *2.2 × 10^−16^

*Note*: Differences tested using a two-sided Mann-Whitney U test.

If disease genes are better studied, and better studied genes are involved in greater numbers of PPIs in each of the HD datasets, then study bias may at least partially explain why disease genes are involved in more interactions than non-disease genes in the HD datasets, but not the HF dataset. To determine whether disease genes are better studied, we used the number of PubMed-indexed publications related to each gene in gene2pubmed (NCBI Resource Coordinators, 2016) (downloaded 11 August 2017) as a measure of how well studied each gene is. Disease genes tend to be related to greater numbers of publications (median 59 publications) than non-disease genes (median 13 publications, *P* < 2.2 × 10^−16^, Wilcoxon rank sum test) indicating that they are better studied. Better-studied genes are involved in more PPIs than less-well-studied genes in each of the PPI datasets (Table 2), although this difference is much greater in the HD datasets than in the HF dataset. The fact that disease genes tend to be better studied than non-disease genes, and that better-studied genes are involved in more PPIs than less-well-studied genes in the HD datasets, may partly explain why disease genes are observed as being involved in more PPIs than non-disease genes in the HD datasets. Study bias may therefore at least partially account for the differences in the numbers of PPIs that disease and non-disease genes are involved in in the HD datasets.

**Table 2.** Numbers of PPIs better and less-well-studied genes are involved in

Database	Better-studied genes		Less-well-studied genes		Difference
	Mean n. PPIs	Median n. PPIs	Mean n. PPIs	Median n. PPIs	
BioGRID	37.8	18.0	7.2	3.0	*P* < 2.2 × 10^−16^
HI-II-14	6.8	2.0	6.2	2.0	*P* = 0.013
HPRD	15.2	8.0	3.4	2.0	*P* < 2.2 × 10^−16^
IntAct	26.4	12.0	6.5	3.0	*P* < 2.2 × 10^−16^

*Note*: Better and less-well-studied genes are defined as those in the top and bottom thirds of genes ranked by the number of related publications in gene2pubmed. Differences tested using a two-sided Mann-Whitney U test.

### 3.2 Bias in network-based gene prioritization methods

We measured the correlations between gene scores computed by PhenoRank and three other prioritization methods, and features of the data used by each method, to determine whether the methods are biased by the numbers of data associated with each gene. Gene scores computed by DADA, EXOMISER-Walker and PRINCE are positively correlated with the network degree of each gene and the numbers of associated sources of phenotype data (i.e. the number of human diseases and model organism mutants associated with each gene), suggesting that these methods score genes more highly if they are associated with more data (Table 3). The scores computed by PhenoRank-NoSimulation are similarly correlated with network degree and the number of associated sources of phenotype data, whilst the gene scores computed by PhenoRank-Simulation are less strongly correlated with these data features, indicating that the use of simulated sets of phenotype terms ensures that PhenoRank is less biased by data availability.

**Table 3.** Correlations between the gene scores computed by each method and features of the data used by each method

Data feature	PhenoRank-Simulation	PhenoRank-NoSimulation	DADA	EXOMISER	PRINCE
Network degree of each gene	−0.04	0.91	0.64	0.46	0.47
Number of sources of phenotype data	−0.03	0.21	0.32	0.18	0.26
Number of annotating phenotype terms	−0.04	0.13	*NA*	0.01	*NA*
Difference in the number of annotating phenotype terms	−0.01	−0.03	*NA*	−0.05	*NA*
Ontology depth of annotating phenotype terms	−0.03	0.18	*NA*	0.02	*NA*

*Note*: DADA and PRINCE do not quantify phenotypic similarity using terms from phenotype ontologies, and therefore correlations involving phenotype terms were not measured for these methods. Correlations measured using Spearman’s rank correlation coefficient.

Gene scores computed by PhenoRank-NoSimulation also correlate with the number of phenotype terms annotating the sources of phenotype data associated with each gene, and the ontology depth of these phenotype terms (Table 3). This suggests that PhenoRank-NoSimulation scores genes more highly if the human diseases and mouse mutants associated with the gene are annotated with greater numbers of terms from phenotype ontologies. Differences between the number of phenotype terms annotating the query disease, and the numbers of phenotype terms annotating gene-associated human diseases and mouse mutants, do not correlate with computed gene scores, suggesting that this data feature is not a major source of bias. Gene scores computed by PhenoRank-Simulation are less strongly correlated with the number of annotating phenotype terms and the ontology depth of these terms, indicating that the use of simulated sets of phenotype terms reduces bias introduced by how well human diseases and mouse mutants are annotated with phenotype terms. Despite EXOMISER-Walker also using phenotype terms to score genes, the gene scores computed by EXOMISER-Walker are not strongly correlated with the numbers of annotating phenotype terms, or the ontology depths of these terms, possibly reflecting differences in the EXOMISER-Walker and PhenoRank-NoSimulation methodologies.

### 3.3 Evaluation of method performance

**Fig. 2.**
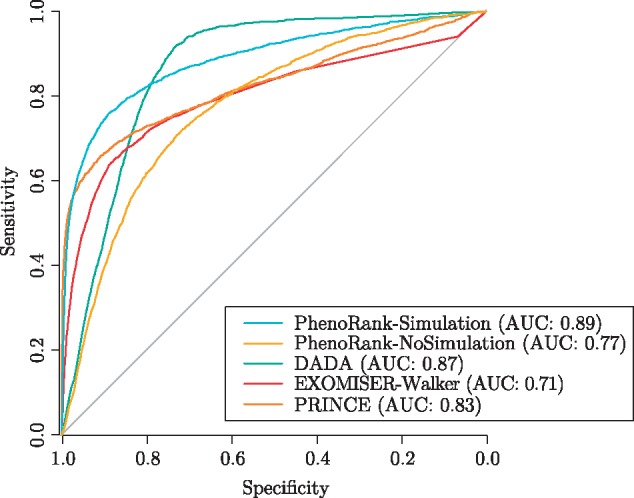
Performances of PhenoRank-Simulation, PhenoRank-NoSimulation, DADA, EXOMISER-Walker and PRINCE in leave-one-out cross-validation

We used leave-one-out cross-validation to evaluate the performances of PhenoRank-Simulation, PhenoRank-NoSimulation, DADA, EXOMISER-Walker and PRINCE (Fig. 2). Using simulated sets of phenotype terms improves the performance of PhenoRank (PhenoRank-Simulation AUC = 0.89, PhenoRank No-Simulation AUC = 0.77, *P *< 2.2 × 10^−16^, two-sided DeLong’s method). Reducing the bias of PhenoRank towards genes with more available data therefore also improves its performance. PhenoRank-Simulation outperforms DADA (AUC = 0.87, *P *< 2.2 × 10^−16^), EXOMISER-Walker (AUC = 0.71, *P *< 2.2 × 10^−16^) and PRINCE (AUC = 0.83, *P *< 2.2 × 10^−16^). Whilst DADA is the method with overall performance most similar to PhenoRank-Simulation, it performs much worse than PhenoRank at higher specificities, with PhenoRank-Simulation and DADA achieving sensitivities of 87 and 47% at 90% specificity, respectively. DADA however outperforms PhenoRank-Simulation at lower specificities, with PhenoRank-Simulation and DADA achieving sensitivities of 92 and 98% at 50% specificity, respectively.

**Fig. 3.**
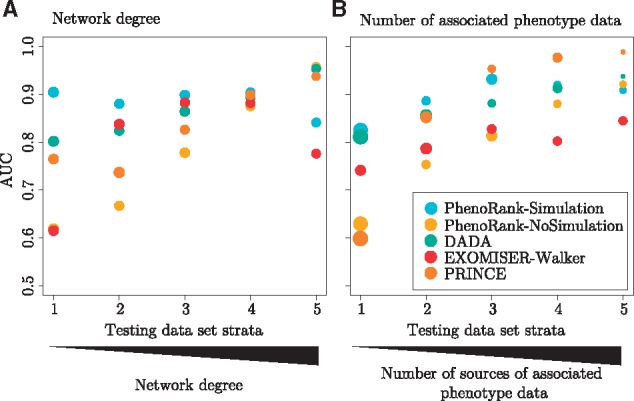
Method performance when applied to genes with different numbers of associated data. For each method, the testing dataset of 2708 disease–gene associations was stratified based on (**A**) the network degree of each gene and (**B**) the number of sources of phenotype data associated with each gene, and leave-one-out cross-validation completed. The size of each circle represents the numbers of disease–gene associations in the testing dataset strata

The bias of DADA, EXOMISER-Walker and PRINCE towards genes with more available data may affect their ability to effectively prioritize genes associated with fewer available data. To test this, we stratified the cross-validation procedure by the PPI network degree of each gene and the number of phenotype data associated with each gene. The set of 2708 disease–gene associations used in method performance evaluation was split into five strata based on the degree of the gene in each disease–gene association, and five strata based on the number of phenotype data associated with the gene in each disease–gene association. Cross-validation was then run using each of the strata (Fig. 3, Supplementary Table S6). Each method uses different data and how the disease–gene associations were stratified was therefore not the same for each method. DADA, EXOMISER-Walker and PRINCE perform better when applied to genes with greater degrees and associated with more phenotype data, reflecting their biases towards genes associated with greater numbers of data. Whilst the performance of PhenoRank-NoSimulation is similarly affected by the numbers of associated data, PhenoRank-Simulation performs more consistently across the strata, demonstrating that the use of simulated sets of phenotype terms reduces the effect of data availability on method performance. PhenoRank may therefore be especially useful when prioritizing genes for which fewer data are available. 

Genes associated with diseases of different classes are associated with different numbers of data (Supplementary Table S7) possibly reflecting how well studied different disease classes are. To determine whether the performances of PhenoRank-Simulation, DADA, EXOMISER-Walker and PRINCE vary between disease classes, we ran the cross-validation procedure using disease–gene associations stratified by disease class (Supplementary Fig. S7, Supplementary Table S8). PhenoRank-Simulation was the best performing method in 20 of the 30 disease classes, DADA in 3 disease classes, EXOMISER-Walker in 2 disease classes and PRINCE in 6 disease classes. These performance differences may be influenced by the biases exhibited by the methods. PhenoRank-Simulation outperforms DADA, EXOMISER-Walker and PRINCE in the Monogenic Disease and Integumentary System Disease classes (*P *<* *0.05), but PRINCE outperforms PhenoRank in the Cancer class (*P *<* *0.05). This may reflect the fact that the mean degree of genes in the PPI network used by PRINCE is higher for genes associated with diseases in the Cancer class (69.8), than genes associated with diseases in the Monogenic Disease (42.8) and Integumentary System Disease (29.6) classes.

In the datasets used by PhenoRank-Simulation, 3713 human protein-coding genes are associated with at least one human disease, 8607 with mouse phenotype data and 9618 with either. To determine whether the performance of PhenoRank-Simulation is improved by using data from both species, we ran the cross-validation procedure using only human disease data and only mouse phenotype data. PhenoRank-Simulation performs better when using both human and mouse data (AUC = 0.89), than when using only human data (AUC = 0.85, *P *< 2.2 × 10^−16^) and only mouse data (AUC = 0.80, *P *< 2.2 × 10^−16^) demonstrating that PhenoRank successfully integrates data from the two species.

### 3.4 Application of PhenoRank to genes in loci associated with rheumatoid arthritis

We prioritized likely causal genes in loci identified in a GWAS of rheumatoid arthritis (Okada *et al.*, 2014) using PhenoRank. Candidate genes in the loci were identified by first selecting single nucleotide polymorphisms (SNPs) in linkage disequilibrium with the lead SNP (*r*^2^ > 0.05) in European populations (Machiela and Chanock, 2014). Regions spanning these SNPs were then defined and all genes whose protein-coding regions at least partially overlap these regions were considered candidates. Loci containing a gene already known to be associated with rheumatoid arthritis were not considered. PhenoRank was then run using rheumatoid arthritis as the input disease and the scores of the genes in these loci extracted from the generated results file. Four genes (*PADI2*, *SYT7*, *LGALS1* and *PLCL2*) in the identified loci were implicated by PhenoRank as being involved in rheumatoid arthritis development (*P *<* *0.05, after within-locus correction for multiple testing, Supplementary Table S9). None of these four genes are associated with any human disease in the data used by PhenoRank, although inflammatory and autoimmune phenotypes have been observed in mutants of their mouse orthologs, including ‘increased susceptibility to experimental autoimmune encephalomyelitis’ and ‘abnormal adaptive immunity’ (Supplementary Table S9). These genes also interact with genes with immune system functions, including *CD4* and *CD8A* (Zhu *et al.*, 2010). It is for these reasons that PhenoRank identifies them as being potential candidates. Some of these genes have been previously implicated in autoimmune disease: *PADI2* expression has been demonstrated to correlate with arthritis severity in mice (Johnsen *et al.*, 2011), *LGALS1* damages cartilage via inflammation in osteoarthritis (Toegel *et al.*, 2016) and *PLCL2* has been associated with systemic sclerosis (Arismendi *et al.*, 2015).

## 4 Conclusions

It has been suggested that the proteins involved in greater numbers of interactions may be less able to tolerate mutations, as a greater proportion of their sequence may be required to facilitate the interactions, thereby increasing the likelihood that they are disease-associated (Yates and Sternberg, 2013). In this study, we show that whilst the protein products of disease genes tend to be involved in greater numbers of PPIs in HD datasets, this may be at least partly a result of them being better studied. PPI networks generated through high-throughput approaches, which are less susceptible to study bias, currently cover only a small proportion of the PPIs thought to occur in cells (Rolland *et al.*, 2014). It may not be possible to determine whether the number of interactions a protein is involved in affects the likelihood of it being disease-associated until we have a more comprehensive, accurate and unbiased map of the interactome.

Genes associated with diseases of difference classes tend to be involved in different numbers of PPIs (Supplementary Table S7). In the PPI network used by PhenoRank, genes involved in cancer have a mean degree of 99.5, whilst genes involved in inherited metabolic disorders have a mean degree of only 18.2. The better characterization of pathways involved in cancer may at least partly explain this difference. The differing performances of PhenoRank, DADA, EXOMISER-Walker and PRINCE across disease classes suggests that a user should consider how well studied a disease is when selecting a prioritization method. A method that performs well when more data are available, such as DADA, EXOMISER-Walker or PRINCE, may be more suitable for studying diseases with more available data, whilst methods that are less biased by data availability, such as PhenoRank, may be more suitable for studying diseases with few available data.

Gillis and Pavlidis (2012) describe study bias in PPI networks in relation to predicting gene function. They suggest that genes involved in more interactions may represent highly studied genes, and that these genes may be more open to the accumulation of false positive interactions as a result of this. If highly studied genes are involved in more false positive interactions, then this may partially explain why the use of simulated sets of disease phenotype terms improves PhenoRank performance, as using this simulation-based approach reduces the score of high-degree genes, thereby reducing the influence of interactions that are more likely to be false positives.

The gene scores computed by PhenoRank-NoSimulation, DADA, EXOMISER-Walker and PRINCE most strongly correlate with the degree of the genes in the networks used by the methods. This suggests that differences in PPI network degree is a major source of bias affecting these methods. Whilst DADA adjusts for the degree of candidate genes in PPI networks in order to better prioritize genes of low degree, it is still biased towards genes of high degree. This is likely because DADA employs a ‘uniform scoring strategy’, in which raw and degree-adjusted gene scores are combined to produce the final gene ranking.

While PhenoRank was developed to prioritize genes in disease-associated loci, the method could be extended to prioritize disease variants. Methods such as EXOMISER and eXtasy (Sifrim *et al.*, 2013) have demonstrated that the integration of a variant effect predictor with gene-level prioritization can aid in pathogenic variant identification. The use of a method that is less biased towards genes for which more data are available, such as PhenoRank, alongside a variant effect predictor may allow for the more effective prioritization of variants in genes that are less well studied.

Whilst existing network-based gene prioritization methods are biased toward genes for which more data are available, the use of simulated sets of phenotype terms ensures that PhenoRank is not similarly biased. Although high-throughput phenotypic screens are being completed (Brown and Moore, 2012), many data sources are still likely to be influenced by study bias. Approaches similar to the simulated sets of phenotype terms used by PhenoRank could be incorporated into existing prioritization methods, such as DADA, PRINCE and EXOMISER, to reduce the influence of study bias.

## Funding

This work was supported by the British Heart Foundation PhD studentship (AJC) and The Wellcome Trust ref WT/104955/Z/14/Z (AD).

*Conflict of Interest:* MJES is a Director and shareholder in Equinox Pharma Ltd., which uses bioinformatics and chemoinformatics in drug discovery research and services.

## Supplementary Material

Supplementary Data
