# Supplementary material for: PhenoRank: reducing study bias in gene prioritization through simulation
Source: Bioinformatics. 2018 Jan 17;34(12):2087–95. doi: 10.1093/bioinformatics/bty028 (PMC5949213; doi:10.1093/bioinformatics/bty028)
Supplement: Supplementary Data [file bty028_supp.zip › bty028-suppl_data/Supplementary Figures.pptx]

## Slide 1
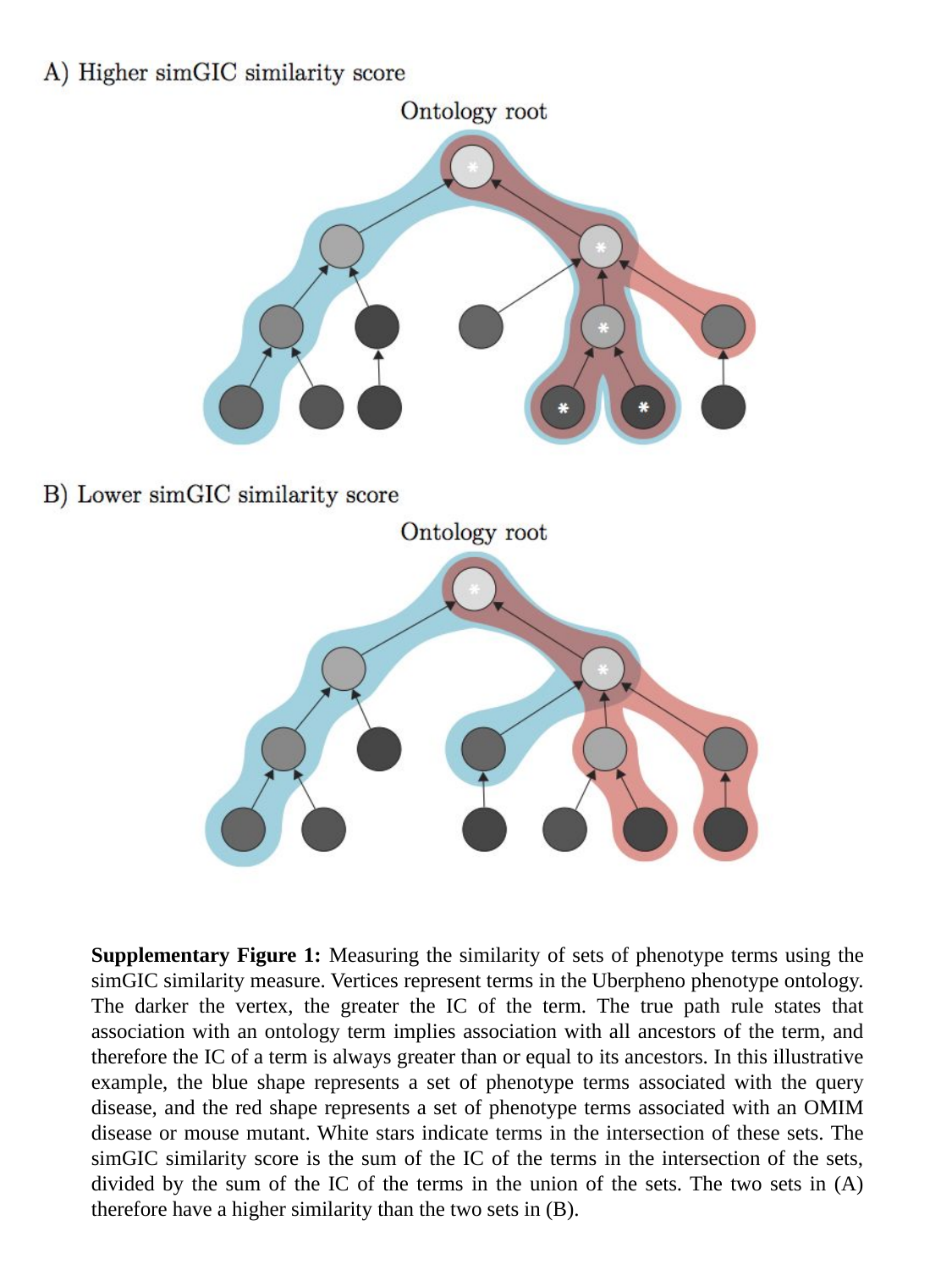

Supplementary Figure 1: Measuring the similarity of sets of phenotype terms using the simGIC similarity measure. Vertices represent terms in the Uberpheno phenotype ontology. The darker the vertex, the greater the IC of the term. The true path rule states that association with an ontology term implies association with all ancestors of the term, and therefore the IC of a term is always greater than or equal to its ancestors. In this illustrative example, the blue shape represents a set of phenotype terms associated with the query disease, and the red shape represents a set of phenotype terms associated with an OMIM disease or mouse mutant. White stars indicate terms in the intersection of these sets. The simGIC similarity score is the sum of the IC of the terms in the intersection of the sets, divided by the sum of the IC of the terms in the union of the sets. The two sets in (A) therefore have a higher similarity than the two sets in (B).

## Slide 2
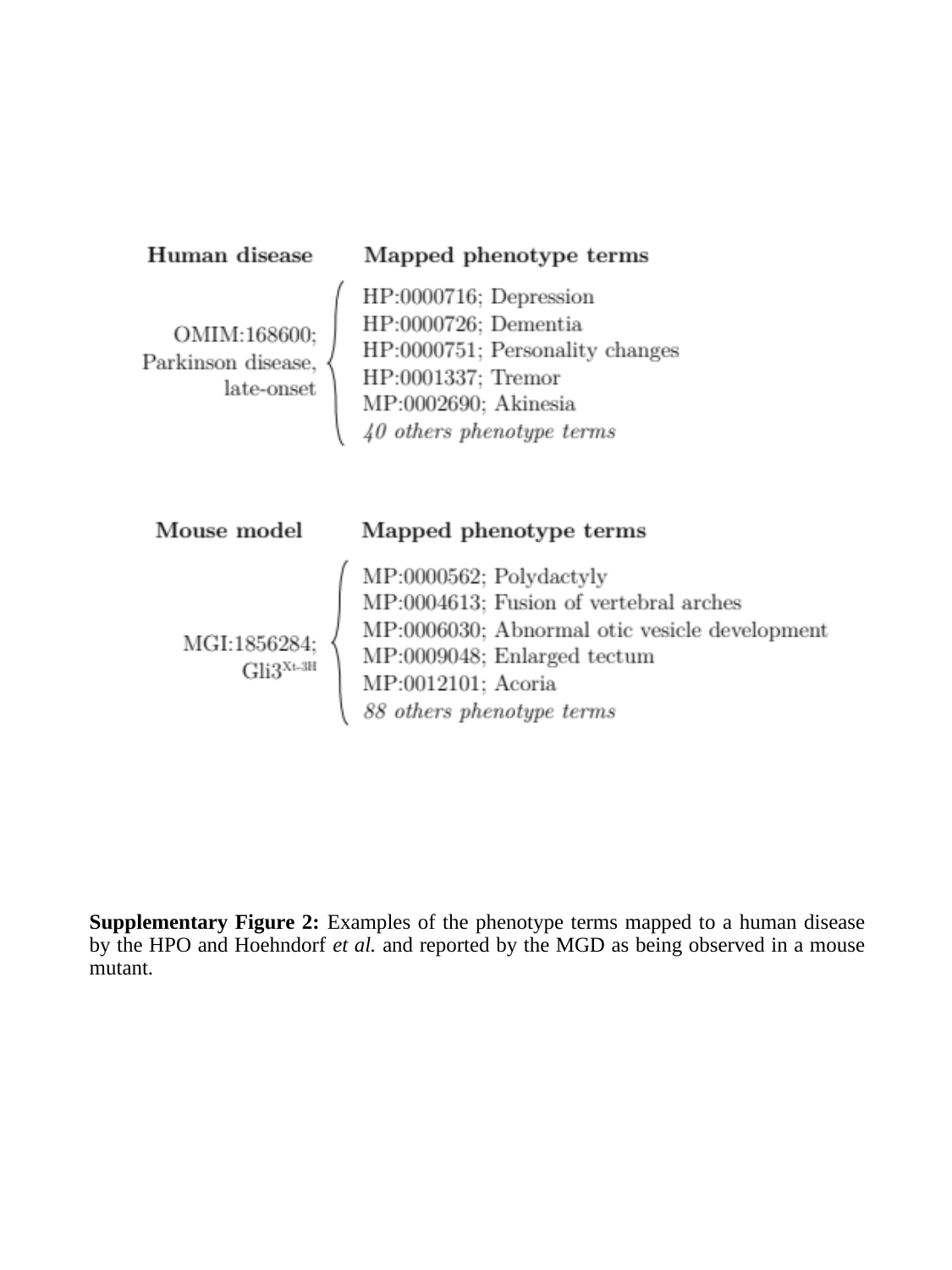

Supplementary Figure 2: Examples of the phenotype terms mapped to a human disease by the HPO and Hoehndorf et al. and reported by the MGD as being observed in a mouse mutant.

## Slide 3
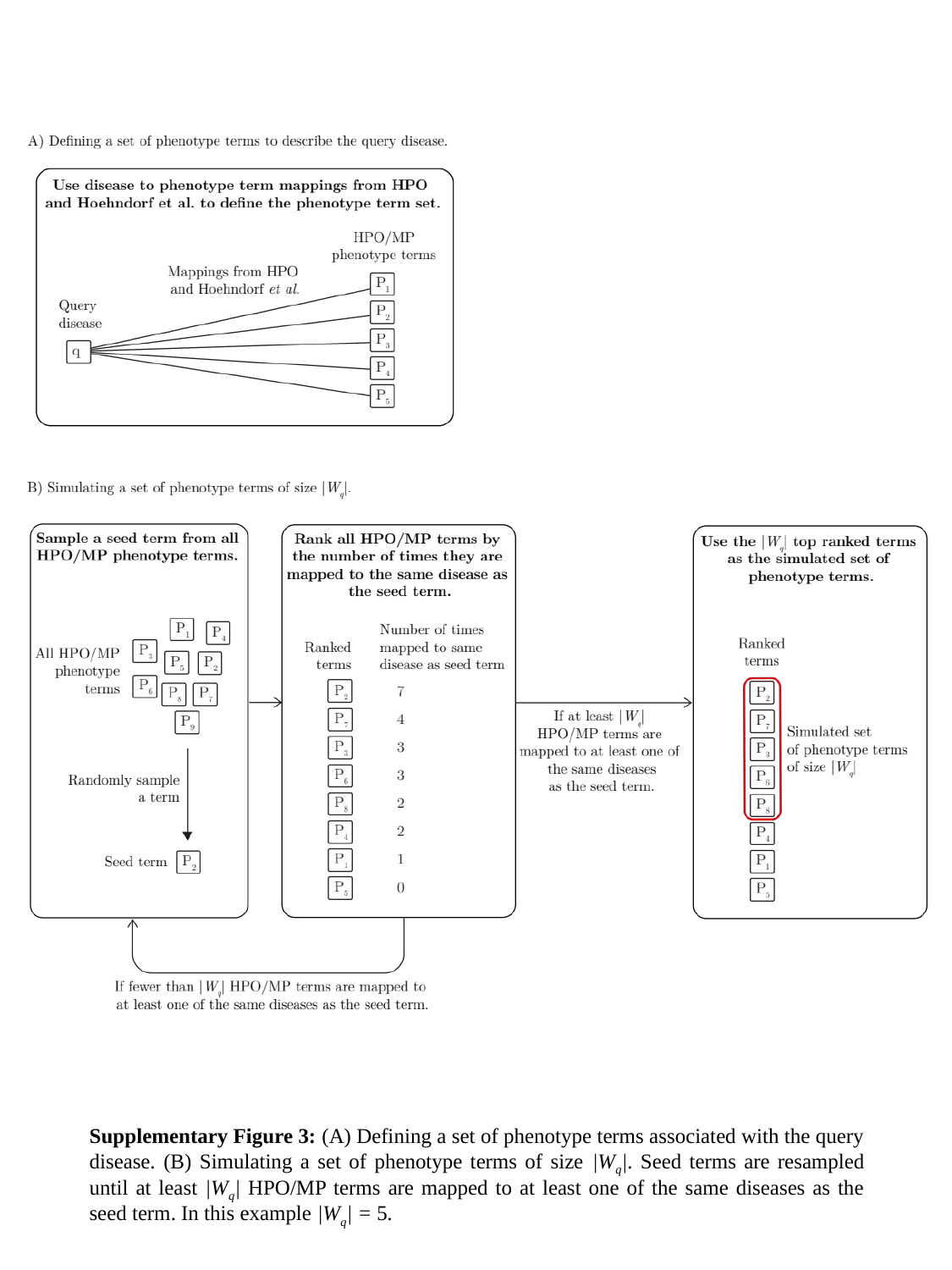

Supplementary Figure 3: (A) Defining a set of phenotype terms associated with the query disease. (B) Simulating a set of phenotype terms of size |Wq|. Seed terms are resampled until at least |Wq| HPO/MP terms are mapped to at least one of the same diseases as the seed term. In this example |Wq| = 5.

## Slide 4
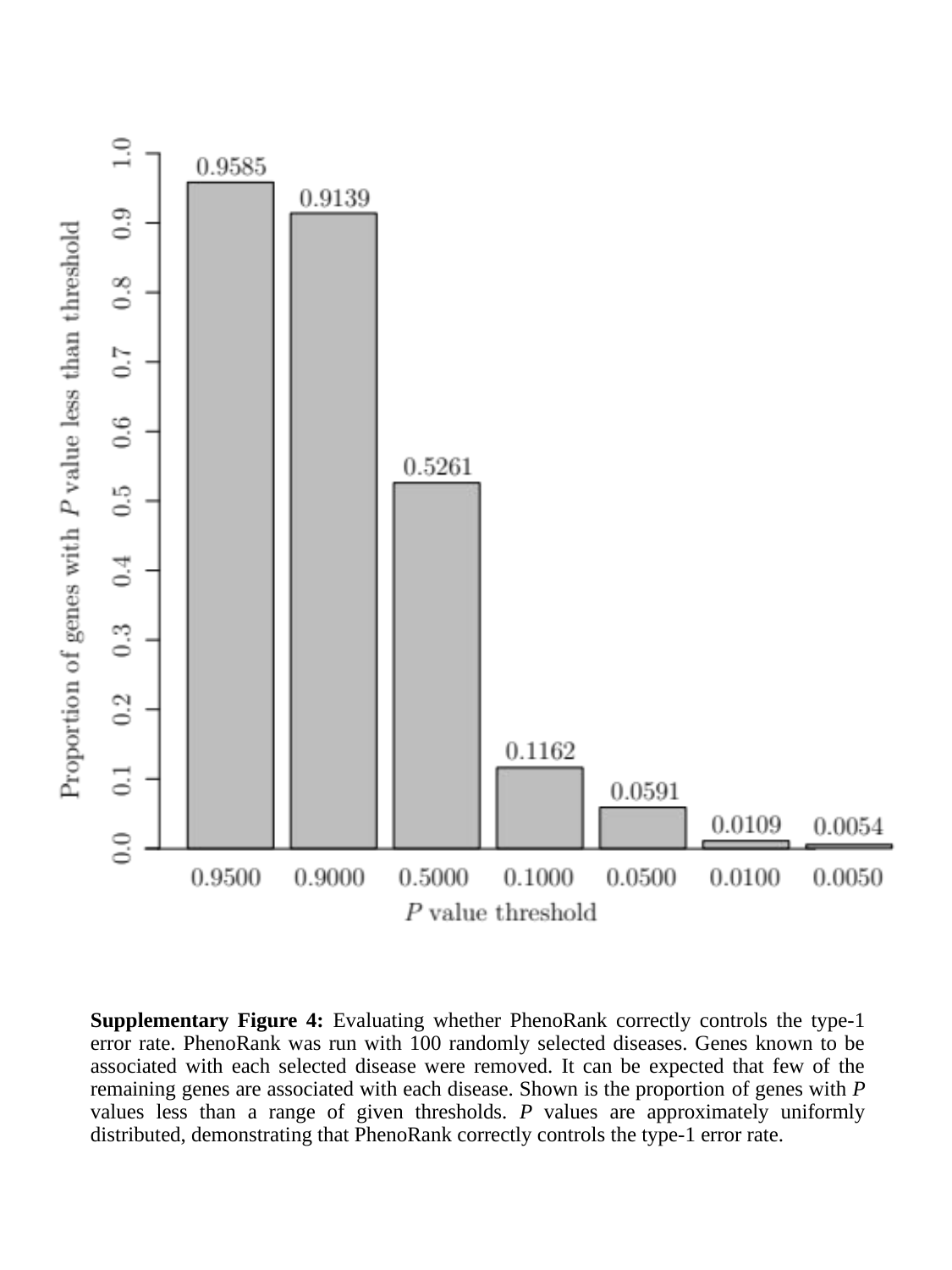

Supplementary Figure 4: Evaluating whether PhenoRank correctly controls the type-1 error rate. PhenoRank was run with 100 randomly selected diseases. Genes known to be associated with each selected disease were removed. It can be expected that few of the remaining genes are associated with each disease. Shown is the proportion of genes with P values less than a range of given thresholds. P values are approximately uniformly distributed, demonstrating that PhenoRank correctly controls the type-1 error rate.

## Slide 5
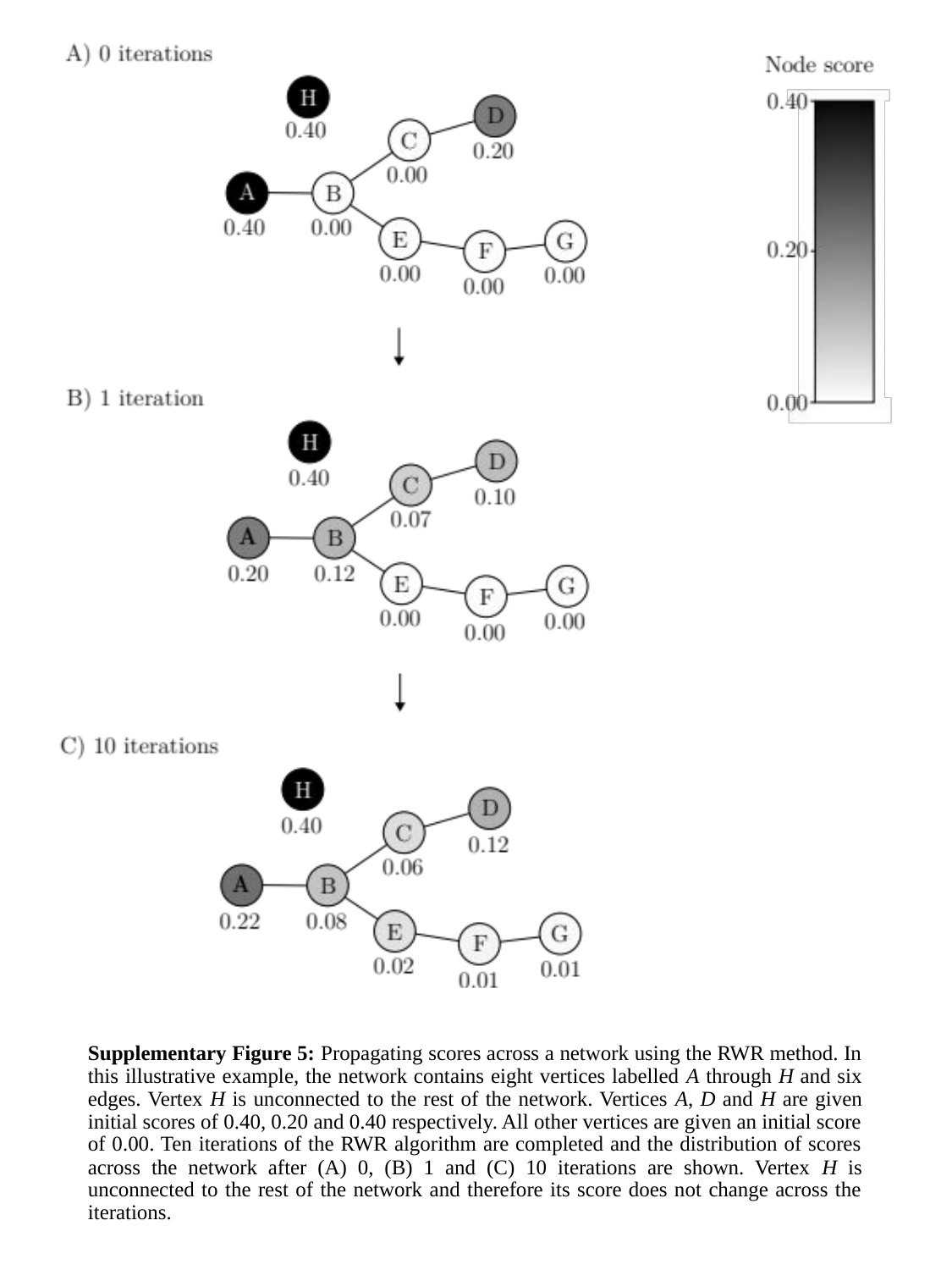

Supplementary Figure 5: Propagating scores across a network using the RWR method. In this illustrative example, the network contains eight vertices labelled A through H and six edges. Vertex H is unconnected to the rest of the network. Vertices A, D and H are given initial scores of 0.40, 0.20 and 0.40 respectively. All other vertices are given an initial score of 0.00. Ten iterations of the RWR algorithm are completed and the distribution of scores across the network after (A) 0, (B) 1 and (C) 10 iterations are shown. Vertex H is unconnected to the rest of the network and therefore its score does not change across the iterations.

## Slide 6
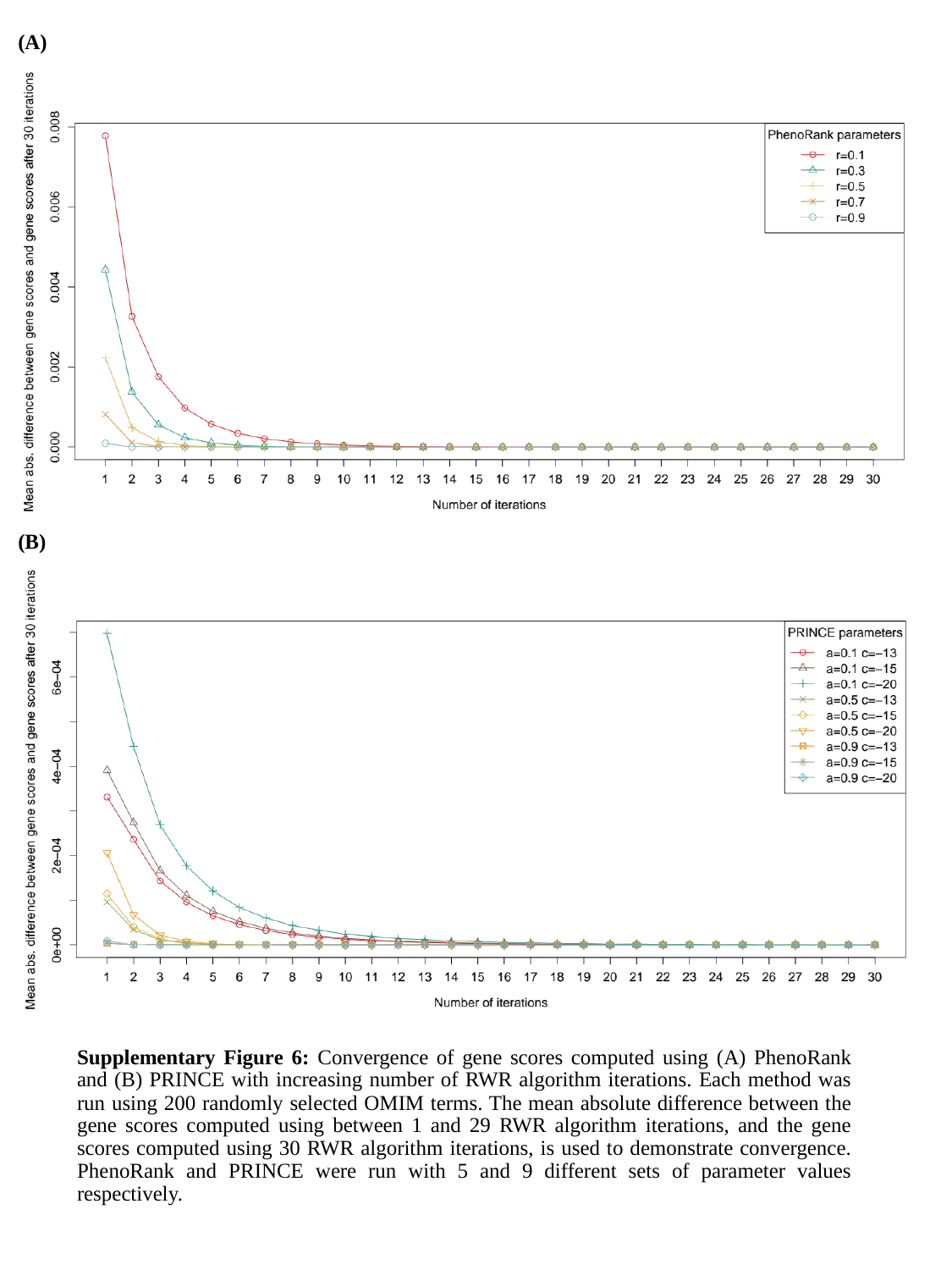

(A)
(B)
Supplementary Figure 6: Convergence of gene scores computed using (A) PhenoRank and (B) PRINCE with increasing number of RWR algorithm iterations. Each method was run using 200 randomly selected OMIM terms. The mean absolute difference between the gene scores computed using between 1 and 29 RWR algorithm iterations, and the gene scores computed using 30 RWR algorithm iterations, is used to demonstrate convergence. PhenoRank and PRINCE were run with 5 and 9 different sets of parameter values respectively.

## Slide 7
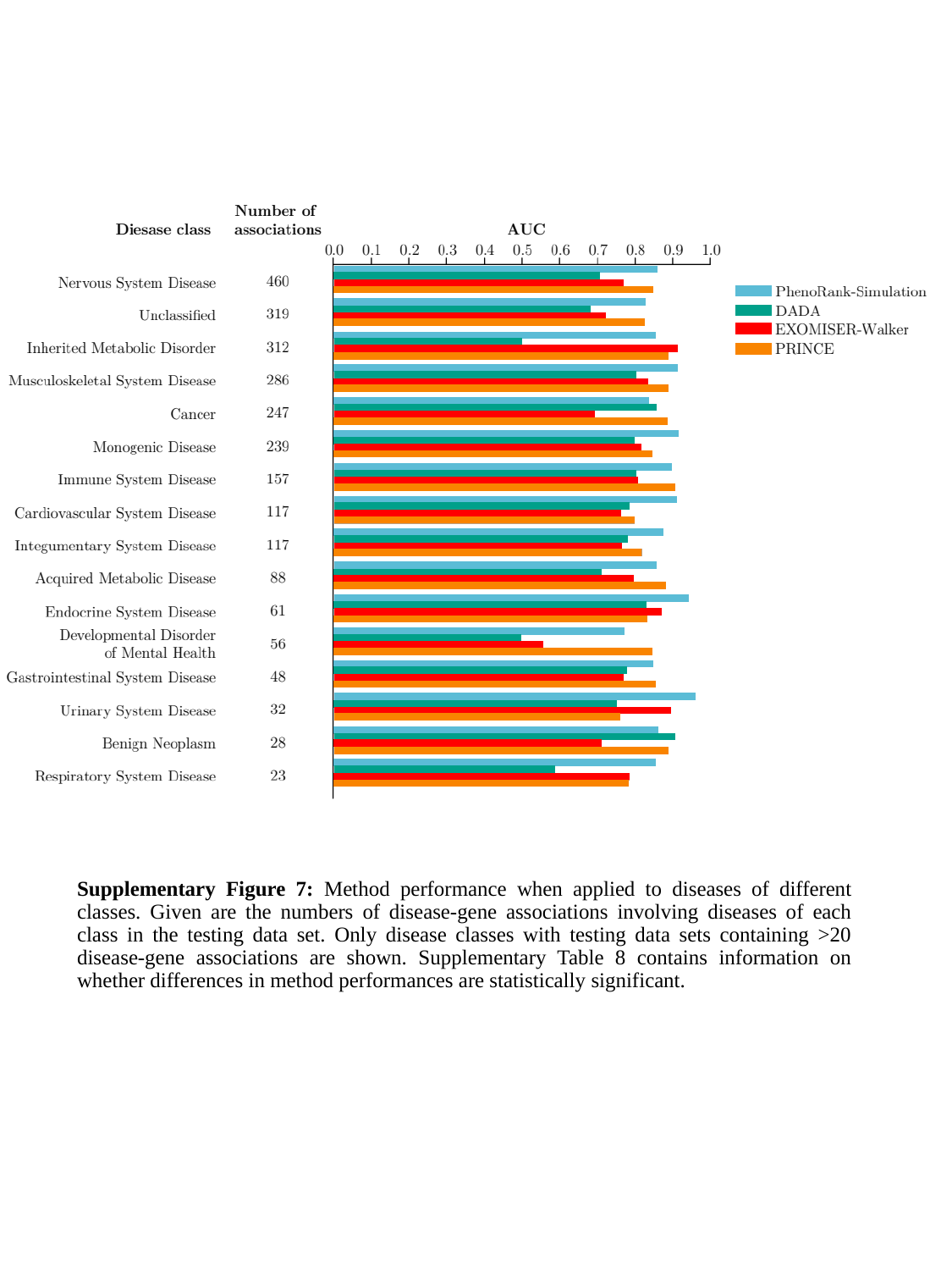

Supplementary Figure 7: Method performance when applied to diseases of different classes. Given are the numbers of disease-gene associations involving diseases of each class in the testing data set. Only disease classes with testing data sets containing >20 disease-gene associations are shown. Supplementary Table 8 contains information on whether differences in method performances are statistically significant.
